# Nonequilibrium Theory for Adaptive Systems in Varying Environments

**Published:** 2025-06-23

**Authors:** Ying-Jen Yang, Charles D. Kocher, Ken A. Dill

## Abstract

Biological organisms thrive by adapting to their environments, which are often unpredictably changeable. We apply recent results from nonequilibrium physics to show that organisms' fitness parses into a static generalist component and a nonequilibrium tracking component. Our findings: (1) Environmental changes that are too fast or too small are not worth tracking. (2) Well-timed anticipatory tracking enhances fitness in coherent environments. (3) We compute and explain the optimal adaptive strategy for a system in a given environment, such as bet hedging or phenotypic memory. Conversely, (4) We compute and explain the optimal way for an environment to control a given system, for example for designing drug regimens to limit the growth of pathogens. By connecting fitness, adaptive strategy, and environmental variability, this work provides the foundations for a generic physical theory of adaptivity.

We seek a generic theory for adaptive dynamics. We consider the dynamics of a system, like a biological organism, that has some *fitness* based on its ability to track dynamical change in its environment. The ability to adapt to varying environments is fundamental for biological survival and success [1–3], prompting extensive theoretical [4–8] and experimental [9–12] investigation into organisms' adaptive strategies. These strategies can be broadly classified into two types: (1) tracking strategies, where the organism senses the environment and shifts its phenotype or internal state according to the current [13, 14] or anticipated [15–20] conditions, and (2) non-tracking strategies (which we call "generalism" in the broad sense), including the "pure" generalist (a single robust phenotype that does not switch) [21, 22] and stochastic bet-hedging (random, environment-independent switching between phenotypes) [23–27]. Previous work has analyzed and computed optimal strategies for given environmental scenarios [4, 28–31] and revealed (phase) transitions to distinct optimal states in different environmental settings [8, 32–34]. However, identifying the optimal strategy alone offers rather limited guidance on the underlying mechanisms and how a suboptimal organism can adjust to improve long-term growth. Knowing where a summit is does not inherently reveal the trail to reach it. We present a general theory of adaptivity which can compute optimized situations and routes to achieve them.

To address this gap, we here derive a useful relationship between the long-term growth rate (population fitness) of an adaptive population, the dynamics of its environment, and the population's strategy. A recent framework of nonequilibrium statistical physics [35, 36] gives a rigorous way to decompose fitness into two distinct components: a baseline non-tracking term quantifying the level of generalism, plus the gain from tracking the environment:

$$\text{fitness} = \text{generalism} + \text{tracking}. \tag{1}$$

Here, we give quantitative applications that show how organisms can optimize their matching to environmental dynamics. We give two examples of a system adapting to match an environment's dynamics: stochastic phenotype switching rates [4, 7] and phenotypic memory [33]. And for the opposite task of controlling an environment to affect the fitness of a given a system – for example in drug delivery strategies to kill potentially drug-resistant pathogens – we compute optimized stochastic or periodic one-drug on-off switching.

Our decomposition of fitness aligns with the general equilibrium-nonequilibrium decompositions widely used in stochastic thermodynamics [37–42]. It shows explicitly that tracking necessarily involves nonequilibrium (NEQ) probability fluxes, clarifying the fundamental connection between time irreversibility and adaptive dynamics [43–48]. In our examples below, these NEQ probability fluxes can arise from responsive phenotype switching, the combined effect of natural selection and bet-hedging, and/or anticipatory phenotype switching.

**How well does a system synchronize with its environment?** First, we derive a basic relationship for how well a system is able to follow changes in its environment. Say we have any general system that takes on different states $\{x\}$ sitting in an environment that takes on different states $\{E\}$. We have a combined space $(E, x)$ of allowed environment-system states. The joint probability of finding the system in state $x$ and the environment in state $E$—or equivalently, of finding the combined environment-system in state $\{(E, x)\}$—is $\pi_{E,x}$. This joint probability describes the long-term time fraction of the joint state $(E, x)$ if a steady-state exists.[1]

* ying-jen.yang@stonybrook.edu
† Now at Memorial Sloan Kettering Cancer Center, New York, NY 10065

[1] The steady state has been called the "red queen" scenario [44, 45, 49, 50], a situation where a species constantly adapts to a changing environment. This term comes from Alice in Wonderland which says that you must always keep running just to "stay in place" (stay alive).

Given the joint distribution, we can compute the marginal distributions ($\pi_E = \sum_x \pi_{E,x}$ and $\pi_x = \sum_E \pi_{E,x}$) and the conditional distribution ($\pi_{x|E} = \pi_{E,x}/\pi_E$) to derive a general *parsing* to the joint probability $\pi_{E,x}$ as a measure of system-environment *synchrony*. See Appendix A of the Supplementary Material (SM) [51] for derivation:

$$\pi_{E,x} = \pi_E\, \pi_x + \sum_{E'(\neq E)} \pi_E\, \pi_{E'}\, (\pi_{x|E} - \pi_{x|E'}). \quad (2)$$

The first term on the right represents a baseline that can be matched with a system independent of the environment (generalism) whereas the second term represents how much a state $x$ is preferred in environment $E$ opposed to other environments $E'$ (tracking). This latter term is the additional contribution from system-environment coupling. To see this, consider a simple two-state sensor aiming to synchronize its state $x = (a, b)$ with a two-state environment $E = (A, B)$, Eq. (2) tells us that concentrated conditional distributions ($\pi_{a|A} > \pi_{a|B}$ and $\pi_{b|B} > \pi_{b|A}$) are needed for the sensor to outperform an independent oscillator (that is, to have $\pi_{A,a} > \pi_A\, \pi_a$ and $\pi_{B,b} > \pi_B\, \pi_b$).

This expresses how tracking affects synchrony. This synchrony parsing is valid for any adaptive dynamics that reaches a joint dynamical steady-state under a varying environment. Below, we now show that sustaining such nonzero conditional distribution differences requires nonequilibrium probability fluxes.

**To track its environment, a system requires nonequilibrium fluxes.** To relate tracking to nonequilibrium flux, we suppose that the combined environment-system follows a continuous-time Markov jump process. In each infinitesimal time step d$t$, only two types of Markov transitions are possible: either the system changes $(E, x) \mapsto (E, x')$ with rates $R_{xx'}(E)$ or the environment changes $(E, x) \mapsto (E', x)$ with rates $R_{EE'}(x)$. A simultaneous change of both $E$ and $x$ is higher order, $\mathcal{O}(\mathrm{d}t^2)$, which is negligible in continuous time. This is known as a *bipartite* process [52–54]. At steady state, the total outward net flux from any joint state $(E, x)$ must be zero:

$$0 = \sum_{E'(\neq E)} \pi_{E,x} R_{EE'}(x) - \pi_{E',x} R_{E'E}(x) + \sum_{x'(\neq x)} \pi_{E,x} R_{xx'}(E) - \pi_{E,x'} R_{x'x}(E). \quad (3)$$

If we further assume that the *environment evolves on its own*,[2] *i.e.* all environment transition rates $R_{EE'}(x) = R_{EE'}$ are independent of the system's state, it can then be shown that, in order to get $\pi_{E,x} \neq \pi_E \pi_x$, we must have nonzero system-wise net fluxes (See Appendix B of SM [51] for derivation and discussion):

$$J_{xx'}(E) = \pi_{E,x} R_{xx'}(E) - \pi_{E,x'} R_{x'x}(E) \quad (4a)$$
$$\neq 0, \text{ for some } (x, x', E). \quad (4b)$$

In other words, the *synchrony parsing* in Eq. (2) is related to *nonequilibrium fluxes* as follows:[3]

$$\pi_{E,x} = \pi_E\, \pi_x + [\text{ gain from } J_{xx'}(E)\,]. \quad (5)$$

The system's nonequilibrium net fluxes are needed for an adaptive system's synchrony to be higher than an independent (non-adaptive) system's would be.[4] This is a main result of this paper. The synchrony $\pi_{E,x}$ can be further mapped to the fitness as the long-term growth rate, provided the instantaneous growth rates $g_x(E)$ of system $x$ under environment $E$ are known. The examples below illustrate what insights result from decomposing the adaptive system's fitness into generalist and tracking components.

## EXAMPLE 1: ADAPTING TO A 2-STATE MARKOV SWITCHING ENVIRONMENT

Here, we illustrate how system-environment dynamics parses into generalist and tracking components in a two-state system. Suppose the system can take either of two states $(a, b)$ under a two-state switching environment $(A, B)$. State $a$ is more fit in environment $A$, and state $b$ is more fit in $B$. For a Markov switching environment and system, the joint system-environment state space is a square with four nodes and four edges, as illustrated in Fig. 1(a). This model can describe, for example, a single chemo-sensing receptor in *E coli* in the fast (un)binding limit [55], or—our focus here—the slow growth limit of phenotype-switching individuals in a population. The synchrony parsing in Eq. (2) now becomes

$$\pi_{Aa} = \pi_A\, \pi_a + \pi_A \pi_B (\pi_{a|A} - \pi_{a|B}) \quad (6a)$$
$$\pi_{Ab} = \pi_A\, \pi_b - \pi_A \pi_B (\pi_{b|B} - \pi_{b|A}) \quad (6b)$$
$$\pi_{Ba} = \pi_B\, \pi_a - \pi_A \pi_B (\pi_{a|A} - \pi_{a|B}) \quad (6c)$$
$$\pi_{Bb} = \pi_B\, \pi_b + \pi_A \pi_B (\pi_{b|B} - \pi_{b|A}). \quad (6d)$$

Let the growth rate of $x$ under $E$ be $g_x(E)$; assume it is much slower than all switching rates ($R_{xx';E}$ or $R_{EE'}$) so that,

[2] This is approximately true if the environment is controlled by an agent external to (and using no knowledge from) the system, if the environment is much larger than the system, or if the system feedback is much slower.

[3] We are not able to derive a generic expression for the second term in Eq. (5). But we have shown that this second term must be zero if all $J_{xx';E} = 0$, regardless of if the environment is equilibrium or not (i.e. $J_{EE'} = 0$ or not). This second term is linear to the fluxes $J_{xx'}(E)$ divided by the environmental switching rates $R_{EE}$, in our examples.

[4] See Appendix C of SM [51] for how the system's net fluxes can be further decomposed into cycle fluxes within the system and those involving both system and environment. The former can be understood as the system's intrinsic nonequilibriumness. Appendix C shows that both types of cycle fluxes can contribute to tracking.

for this example only, the population fractions are dominated by the switching and are approximately not impacted by the growth terms. The Long-Term per capita Growth Rate (LTGR) of the phenotype-switching population $N(t)$ can then be parsed as followed:

$$\text{LTGR} = \lim_{t\to\infty} \frac{1}{t} \ln \frac{N(t)}{N(0)} \approx \sum_{Ex} \pi_{Ex}\, g_x(E) \tag{7a}$$

$$= \underbrace{\pi_a \bar{g}_a + \pi_b \bar{g}_b}_{\text{generalism}} + \underbrace{\pi_A \pi_B \left[\pi_{a|A} - \pi_{a|B}\right] \Delta_g}_{\text{tracking}} \tag{7b}$$

where $\bar{g}_x = \pi_A g_x(A) + \pi_B g_x(B)$ is the environment-averaged growth rate of type $x$ and $\Delta_g = [g_a(A) - g_b(A)] + [g_b(B) - g_a(B)]$ is the total growth rate difference between the more and less fit states.

In this example, the *tracking* term is directly related to the *cyclic net probability flux* in the square joint state space. By using $J = \pi_{Ab}R_{AB} - \pi_{Ba}R_{BA} = \pi_{Ba}R_{BA} - \pi_{Aa}R_{AB}$ and denoting the average environment dwell time as $\bar{T}_A = 1/R_{AB}$ and $\bar{T}_B = 1/R_{BA}$, one can show that (See Appendix D of SM [51] for derivation)

$$\pi_{a|A} - \pi_{a|B} = \pi_{b|B} - \pi_{b|A} = \bar{T} \cdot J \tag{8}$$

where $\bar{T} = \bar{T}_A + \bar{T}_B$ is the average environmental cycle period. This gives a distribution-flux parsing to the fitness:

$$\text{LTGR} = \pi_a \bar{g}_a + \pi_b \bar{g}_b + \pi_A \pi_B \,(\bar{T} \cdot J)\, \Delta_g. \tag{9}$$

This fitness parsing gives two implications on how environment, strategy, and fitness are related.

On the one hand, the marginal state distribution $\pi_a$ and cycle flux $J$ are two dynamical features that quantify statistically the levels of "generalism" and "tracking", respectively, in the system. Pure generalism corresponds to $\pi_a = 0$ or 1, and strong tracking is represented by a large flux $J$. Together with two other symmetric flux observables (traffic), $\tau_E = \pi_{Ea}R_{ab;E} + \pi_{Eb}R_{ba;E}$ where $E = A, B$, these four observables $(\pi_a, \tau_A, \tau_B, J)$ uniquely parameterize the four dimensions of the system's dynamics (Fig. 1b), which are the system's four transition rates $R_{xx';E} = (\tau_E \pm J)/(2\pi_{Ex})$—where $\pi_{Ex}$ is determined by $\pi_a$ and $J$ by Eqs. (6) and Eq. (8). This shows that the system has three equilibrium dimensions $(\pi_a, \tau_A, \tau_B)$ and one nonequilibrium dimension parameterized by $J$, and only $\pi_a$ and $J$ affect the fitness directly.[5] The difference $|\tau_A - \tau_B|$ can represent a symmetric coupling between the environment and the system, but such a coupling has no direct contribution

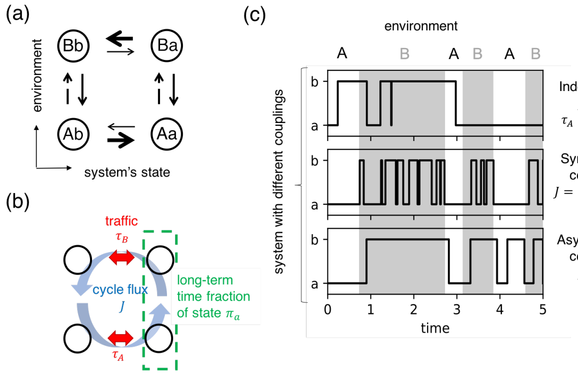

FIG. 1. **The state space, observables, and types of system-environment coupling of a simple adapting individual.** **(a)** The total state space of the two-state system $\{a, b\}$ under a Markov environmental switch $\{A, B\}$. Vertical arrows are environmental transitions and horizontal arrows are system transitions. **(b)** Four statistical observables that fully parameterize the system's dynamics. $\pi_a$ is the long-term time fraction that the system is in $a$ regardless of the environmental state. Traffic $\tau_E$ are the sum of fluxes in both direction under environment $E$. $J$ is the net cycle flux. **(c)** Three types of system-environmental coupling classified by the observables. Net flux $J$ is needed to track the environment and outperform an independent switch in the synchronization task.

to the fitness:[6] asymmetric, nonequilibrium coupling through $J$ is needed for a system to outperform an independent oscillator, as illustrated in Fig. 1(c).

On the other hand, the fitness parsing in Eq. (9) summarizes how environmental statistics or the growth rate profile affects the fitness. We make three points here. (1) If the fitter states $(Aa, Bb)$ are not much fitter, i.e. $\Delta_g$ is small, then the fitness gain from tracking is small as expected. Eq. (9) quantifies that intuition. (2) If the environment switches must faster than the system, i.e. $1/\bar{T} \gg J$, then the fitness gain from tracking diminishes. Faster environments are harder to track, and they switch before you can build up significant benefit. (3) If the environment has a strong bias to one of its states, say $A$, then $\pi_A \to 0 \Rightarrow \pi_A\pi_B \to 0$ and the gain from tracking also diminishes ($\bar{T}$ fixed). Tuning $\pi_a$ is more effective in this case. This example illustrates some implications of parsing the fitness into generalism and tracking components.

## EXAMPLE 2: ACHIEVING OPTIMAL BET-HEDGING

We now go beyond the slow-growth limit of a population in Example 1 to consider a large phenotype-switching

[5] The traffic terms can affect the fitness indirectly. The two traffic terms are the upper bound the net flux, $J \leq \min(\tau_A, \tau_B)$, by their definitions. Thus, raising the traffic terms could give "more room" for the tracking flux to increase the fitness.

[6] Note that if a system has symmetric coupling to the environment, defined as having asymmetric traffic $\tau_A \neq \tau_B$ but zero net flux $J = 0$, then the system and the environment are statistically independent $\pi_{Ex} = \pi_E \pi_x$ but dynamically coupled, since in general $R_{xx';E} = \tau_E/(2\pi_{Ex}) \neq R_{xx';E'} = \tau_{E'}/(2\pi_{E'x})$. Statics and dynamics are fundamentally different, and zero mutual information does not imply no coupling.

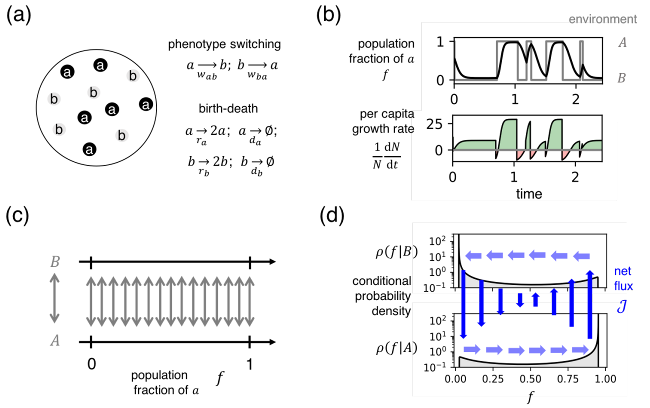

FIG. 2. **The dynamics and total state space for a phenotype-switching population.** **(a)** We consider the population dynamics of two-state individuals with Poisson birth-death and switching. **(b)** The population type fraction (of $a$) tracks the environmental changes and determines the instantaneous per capita growth rate. **(c)** The system-environment total state space consists of two line segments $f \in [0, 1]$, one for each environment $A$ and $B$. The population can jump between two segments at any $f$. **(d)** The (cyclic) net flux over the total state space leads to the long-term conditional probability density $\rho(f|E)$, forming a Flux-Tracking Relation for population dynamics.

population where individuals can reproduce, die, or switch phenotype under a Markov switching environment, as illustrated in Fig. 2(a). The same set of equations could also describe a species that reproduces with mutation (see Appendix E of SM [51]). For simplicity, we assume the reproduction, death, and switching are Poissonian. Then, for a large population under a fixed environment $E$, the dynamics are described by ODEs:

$$\frac{dN_a}{dt} = g_a(E)N_a - w_{ab}(E)N_a + w_{ba}(E)N_b \ , \tag{10a}$$

$$\frac{dN_b}{dt} = g_b(E)N_b + w_{ab}(E)N_a - w_{ba}(E)N_b, \tag{10b}$$

where $g_x = r_x - d_x$ is the net growth rate of type $x$ with reproduction rate $r_x$ and death rate $d_x$, which we presume to be constant and independent of the population size for simplicity.

These equations are commonly used to model phenotypic switching in a population of microbes like *E. coli* [4, 33] or yeast [7]; it is standard to rewrite them in terms of more informative variables. Denoting the total population $N = N_a + N_b$ and the population fraction of the $a$ phenotype $f = N_a/N$, these ODEs become

$$\frac{df}{dt} = f(1-f)\,\Delta_{ab} + (f_w - f)\sigma_w \ , \tag{11a}$$

$$\frac{dN}{dt} = [g_a f + g_b(1-f)]\, N \ . \tag{11b}$$

where $\sigma_w = w_{ab} + w_{ba}$ is the sum of the switching rates, $f_w = w_{ba}/\sigma_w$ is the steady-state phenotype fraction if there's no growth (or death), and $\Delta_{ab} = g_a - g_b$ is the growth rate difference, also known as the selection coefficient. All these parameters depend on the environment. The rewriting shows that the diversification mechanisms of phenotypic switching drive the type fraction $f$ toward $f_w$ with rate $\sigma_w$ whereas natural selection, via the other term of $df/dt$, aims to fixate $f$ toward 0 or 1 with a rate $|\Delta_{ab}|$. The overall dynamics is a balance of the two. Most importantly, it shows that the type fraction $f$ is the sensing dimension that tracks the environmental changes, whereas the $N$ dynamics in Eq. (11b) converts $f$ into the per capita growth rate as the population's average fitness. See Fig. 2(b) for an example trajectory.

**The phenotype fraction determines the Long-Term Growth Rate.** We now derive the fitness parsing for this adaptive population. The LTGR of the total population under a switching environment can be directly related to the long-term average $f$ conditioned on different environments $\mathcal{E}$:

$$\text{LTGR} = \lim_{t\to\infty} \frac{1}{t} \ln \frac{N(t)}{N(0)} \tag{12a}$$

$$= \sum_{\mathcal{E}} \pi_{\mathcal{E}} \left[ g_a(\mathcal{E})\langle f|\mathcal{E}\rangle + g_b(\mathcal{E})(1 - \langle f|\mathcal{E}\rangle) \right] \tag{12b}$$

where $\pi_{\mathcal{E}}$ is the long-term time fraction of environment $\mathcal{E}$ and

$$\langle f|\mathcal{E}\rangle = \lim_{t\to\infty} \frac{\int_0^\infty f(t)\delta_{E(t),\mathcal{E}}dt}{\text{total time in } \mathcal{E}} \tag{13}$$

is the conditional long-term empirical average of $f$ under all epochs in environment $\mathcal{E}$. The term $\delta_{x,y}$ within the integral is a Kronecker delta function, which collapses the integral so that it is only taken over times when the system is in environment $\mathcal{E}$. For Markov switching environments, the conditional average $\langle f|\mathcal{E}\rangle$ can be expressed analytically and computed numerically without ensemble simulations [29–31], which we've used throughout the paper.

We can thus parameterize the system's state space with only the type fraction $f$.[7] With a Markov switching environment $A \leftrightarrow B$, the system-environment joint state space is then composed of two line segments $f \in [0, 1]$, one under each environment $A$ and $B$, as shown in Fig. 2(c).

By computing the long-term average value of $f$,

$$\langle f\rangle = \lim_{t\to\infty} \frac{\int_0^\infty f(t)dt}{\text{total time}} = \pi_A\langle f|A\rangle + \pi_B\langle f|B\rangle, \tag{14}$$

we get the fitness decomposition of this example by rewriting Eq. (12b), which is a direct population generalization of the

[7] This simplification is due to the density independence of the switching and growth rates. For more general population dynamics, such as the case with a finite carrying capacity, the $f$ dynamics will depend on $N$, and we will need both $f$ and $N$ to parameterize the system's state under the varying environment.

small growth case in Eq. (7b) with individual probability $\pi_a$ replaced by average population fraction $\langle f\rangle$:

$$\begin{aligned}\text{LTGR} =&\langle f\rangle \bar{g}_a + \langle 1-f\rangle \bar{g}_b \\ &+ \pi_A\pi_B \left[\langle f|A\rangle - \langle f|B\rangle\right]\Delta_g. \end{aligned} \tag{15}$$

Further denote that $J(f, A)$ is the probability flux in the $f$ direction under environment $A$, as illustrated in horizontal, blue arrows in Fig. 2 (d), and $\bar{J} = \int_0^1 J(f, A)\mathrm{d}f$ is the total net flux. Then, we can relate the tracking term in Eq. (15) to net flux and get a fitness decomposition in the *exact same form* as our earlier case in Eq. (9) (see Appendix F in SM [51] for derivation):

$$\text{LTGR} = \langle f\rangle \bar{g}_a + \langle 1-f\rangle \bar{g}_b + \pi_A\pi_B \left(\bar{T}\cdot\bar{J}\right)\Delta_g. \tag{16}$$

This shows that the relationship among environment, strategy, and fitness in Example 1 also applies to adaptive populations in Example 2.

**An optimization principle for stochastic bet-hedging.** We now discuss how the fitness parsing in Eq. (16) helps explain non-monotonic trends upon altering the system parameters [4, 7, 9, 33]. Bet-hedging is a common and often useful strategy for biological systems to survive varying environments [2]. In the context of a phenotype-switching population, it is often implemented mechanistically through phenotype switching with rates $w_{ab}$ and $w_{ba}$. The population maintains diversity through switching, preventing natural selection from fixing either phenotype (reaching $f = 0$ or $f = 1$). Here, we discuss how such diversification helps the long-term survival of the population in the case where $w_{ab}$ and $w_{ba}$ are independent of the environment, often called *stochastic switching* [4].

Kussell and Leibler have shown that, in the fast growth limit where $g_x(E) \gg$ all $R_{EE'}$ and $w_{xx'}$, the optimal stochastic switching rate equals the environmental switching rate [4], see Fig. 3(a) for an illustration. When the environment is not slow, however, the existence and position of the optimal switching rate depend on both the environment and the growth rate profile [7]. Our fitness decomposition explains why and gives a sufficient condition for the existence of optimal non-zero switching rates.

We've parsed fitness into two parts in Eq. (16): one associated with the long-term average baseline fraction $\langle f\rangle$ (first two terms) and another related to the net flux and environmental tracking (last term). First, we note that the tracking term is always bell-shape-like in the two dimensional space of stochastic switching rate $w$, always attaining a maximum on the interior of the region and never on the boundary. This is due to two distinct features. On the one hand, it vanishes when either of the stochastic switching rates is tiny—causing near fixation because of the selection force $\mathrm{d}f/\mathrm{d}t \approx f(1-f)\,\Delta_{ab}$. On the other hand, when switching rates ($w_{ab}$ and $w_{ba}$) are both large, it screens out the environment-dependent natural selection by $\mathrm{d}f/\mathrm{d}t \approx (f_w - f)\sigma$, also leading to vanishing flux $\bar{J}$.

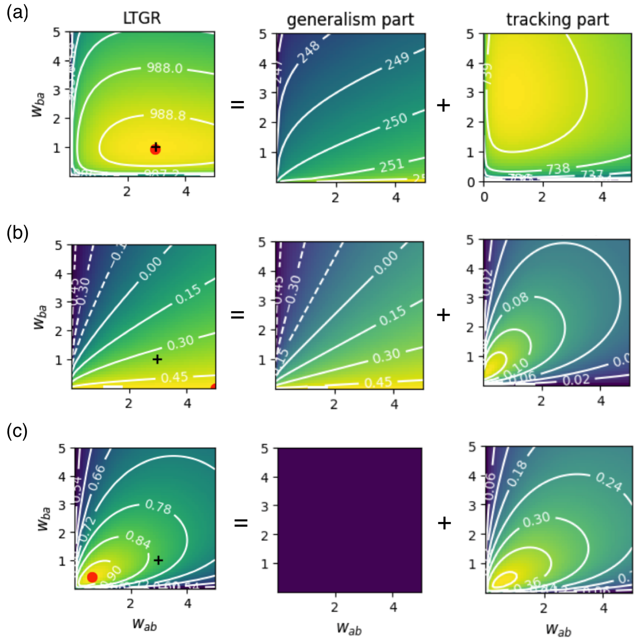

FIG. 3. **Fitness Parsing explains the existence of optimal nonzero stochastic switching rates.** Analyzing the dependence of Long-Term (logarithmic) Growth Rate (LTGR) on the stochastic switching rates $(w_{ab}, w_{ba})$. Black "+" denotes the position where $(w_{ab}, w_{ba}) = (R_{AB}, R_{BA})$ whereas red "o" denotes the optimal $(w_{ab}, w_{ba})$. **(a) Fast growth limit.** The optimal switching rates match the environment's switching rates in this limit. **(b) When the generalist strategy is optimal.** The case where the growth rate is not fast. The optimum LTGR happens at the boundary, with $w_{ba} = 0$. **(c) When tracking as the combined effect of bet-hedging and natural selection is optimal.** This is a case where the environmental average growth rates of the two types are the same: $\bar{g}_a = \bar{g}_b = 0.5$. The middle $\langle f\rangle$ part shows a flat landscape with a uniform value of 0.5. Parameters used are the following: (a) $R_{AB} = 3$, $R_{BA} = 1$, $g_a(A) = 1000$, $g_b(A) = -1000$, $g_a(B) = -1000$, $g_b(B) = 1000$; (b) $g_a(A) = 1$, $g_b(A) = -1$, $g_a(B) = -1$, $g_b(B) = 1$. All other parameters are the same as (a); (c) $R_{AB} = 3$, $R_{BA} = 1$, $g_a(A) = 5$, $g_b(A) = -1$, $g_a(B) = -1$, $g_b(B) = 1$.

Therefore, the tracking contribution is always a bell shape, as illustrated on the right column throughout Fig. 3(a-c).

Yet, the tracking term from NEQ flux is not the full story. The generalism effect of $\langle f\rangle$ can come in and ruin the bell shape contributed by tracking since it maximizes at the boundaries, either $\langle f\rangle = 1$ or 0 (the pure generalist strategy) depending on whether $\bar{g}_a > \bar{g}_b$ or $\bar{g}_a < \bar{g}_b$. The case in Fig. 3(b) is an example. With this and Eq. (16), we can now obtain a sufficient condition that the optimum LTGR occurs at nonzero bet-hedging rates (both $w_{ab} > 0$ and $w_{ba} > 0$): it is when the two environment-averaged growth rates are the same, $\bar{g}_a = \bar{g}_b$. In such a case, the contribution from the first generalism term is a constant $\bar{g}_b$, regardless of the value of $\langle f\rangle$. The changes of the LTGR from tuning $w_{ab}$ and $w_{ba}$ are then solely determined by the flux term, which gives a maximum with nonzero bet-hedging. See Fig. 3(c) for illustration.

Through this analysis, we have demonstrated how the fitness parsing in Eq. (16) explicitly quantifies the two competing effects of the generalism part and the tracking part. We have further shown how these two parts explain the existence and position of the optimal stochastic switching rates. We remark here that the net flux of tracking in this system does not require each individual to track the environment. The population can track the environmental changes by the differential growth in natural selection alone. The stochastic switching is needed to sustain the tracking, preventing the system from fixation.

## EXAMPLE 3: ACHIEVING OPTIMAL PHENOTYPIC MEMORY.

Some systems can *learn and remember* something about their environments. Some microorganisms can sense their environment and switch their phenotypes accordingly, called *responsive switching* [4]. For example, *E. coli* can activate the *lac* operon to metabolize lactose when it is present, and they can turn it off when more preferred nutrients like glucose are available. Using a simple phenomenological population ODE model, Skanata and Kussell showed that there is an optimal responsive switching rate (whose inverse is interpreted as the strength of phenotypic memory) under a stochastic switching environment [33]. Here, we show that fitness parsing can explain the mechanism behind the optimality and give the conditions for its existence.

Suppose $A$ is the lactose environment, $B$ is the glucose environment, $a$ is the phenotype expressing *lac* proteins, and $b$ is the phenotype that does not. Then, following [33] and evaluating Eq. (11a) for the two environments, the equations for the fraction $f$ of type $a$ under $A$ and $B$ are

$$\frac{\mathrm{d}f}{\mathrm{d}t} = f(1-f)h + (1-f)\tau_{\text{on}}^{-1}, \text{ under } A; \tag{17a}$$

$$\frac{\mathrm{d}f}{\mathrm{d}t} = f(1-f)(-c) + (0-f)\tau_{\text{mem}}^{-1}, \text{ under } B \tag{17b}$$

where $\tau_{\text{on}}$ is the time scale to sense and reach high-level *lac* proteins, $\tau_{\text{mem}}$ is the time scale for the degradation of *lac* proteins (phenotypic memory), and the growth rates for the LTGR computation are $g_a(A) = h$, $g_b(A) = 0$, $g_a(B) = g - c$, $g_b(B) = g$, with $g$ being the growth rate under glucose, $h$ being the growth rate of individuals with high *lac* proteins in the lactose environment, and $c$ being the fitness cost for having high *lac* proteins under the glucose environment. Of interest here is how tuning the memory level $\tau_{\text{mem}}$ affects the LTGR.

Eqs. (17) indicate how tuning $\tau_{\text{mem}}$ affects the generalism term and the tracking term in the fitness parsing Eq. (16). A weaker memory (smaller $\tau_{\text{mem}}$) means a faster switching rate $\tau_{\text{mem}}^{-1}$ from $a \mapsto b$ under the glucose environment $B$, leading to a smaller $\langle f \rangle$ and a bigger net flux. Thus, the flux contribution to LTGR will increase with increasing rate $\tau_{\text{mem}}^{-1}$ as shown on the right hand side of Fig. 4. Whether the decay of $\langle f \rangle$ increases or decreases LTGR depends on whether $\bar{g}_a - \bar{g}_b$ is negative or positive, as shown in the middle column of Fig. 4. On the one hand, if $\bar{g}_a > \bar{g}_b$, the decay of $\langle f \rangle$ leads to a decrease in its contribution to the LTGR, and an intermediate maximum in fitness is then possible as shown in Fig. 4(a). On the other hand, if $\bar{g}_a \leq \bar{g}_b$—*i.e.* if the environment-averaged growth rate of having many *lac* proteins is not bigger than having none, as is the case in Fig. 4 (b-c), the contribution of $\langle f \rangle$ is non-decaying, and less memory (larger $\tau_{\text{mem}}^{-1}$) is always better (no intermediate fitness maximum). This example shows principles for optimizing an adaptive system's memory of its environment.

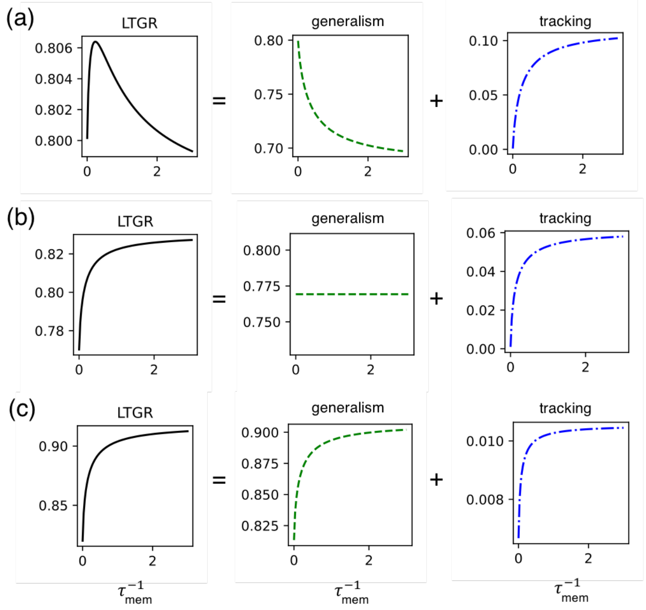

FIG. 4. **Fitness Parsing explains the existence of optimal phenotypic memory.** From (a) to (c), we increase $R_{AB}$ from 2 to 10/3 to 10. The average growth rates of the two environments change from $(\bar{g}_a, \bar{g}_b) = (0.8, 0.\bar{6})$ to $\approx (0.769, 0.769)$ and to $= (0.\overline{72}, 0.\overline{90})$. Optimal phenotype memory can not happen in (b) or (c) when $\bar{g}_a \leq \bar{g}_b$. Other parameters are fixed at $R_{BA} = 1$, $h = 1$, $r_{\text{ON}} = 1$, $g = 1$, $c = 0.3$, following [33].

## EXAMPLE 4: ANTICIPATORY TRACKING ADAPTS TO COHERENT ENVIRONMENTS

So far, we have considered a current system that acts in its current environment. What if the system has the capability to account somehow for past environments? In our Markov modeling above, knowing how long the environment has been in a particular state does not help to predict the timing of the next switch. Here, we consider environments that have some degree of predictability, such as periodicity. Some

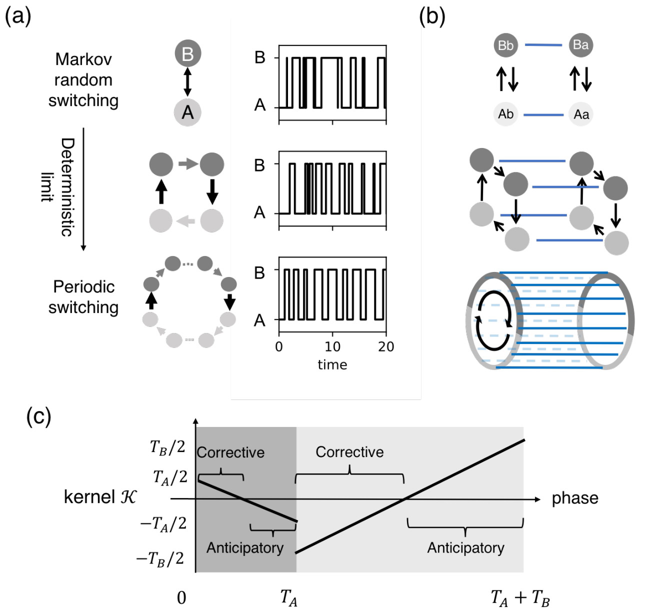

FIG. 5. **State spaces of adaptive systems under a periodic environment and the kernel giving the synchrony gain from fluxes at different environmental phases. (a)** A periodic switching environment can be understood as the limit of infinitely-many intermediate fast Markov transitions. The state space is shown on the left and example trajectories on the right. **(b)** The system-environment total state space for a single-stage Markov switching environment, two-state Markov switching environment, and periodically-switching environment. **(c)** Illustration of the kernel that couples synchrony gain and net fluxes at different phases of the environment.

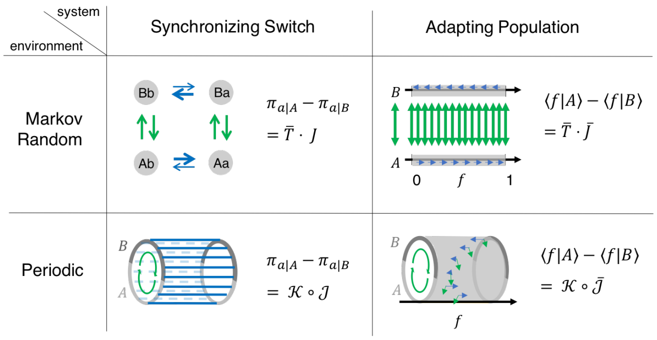

FIG. 6. **The relationship between flux and conditional probability difference is similar for a synchronizing switch and an adapting population under both random and periodic environments.** The column on the left shows flux-tracking relations for a switch under a Markov random environment or periodic environment. The column on the right shows a population of reproducing switches under the two types of environments. Diagrams within each cell are the environment-system state space, with green arrows showing environmental transitions and blue arrows showing system transitions. Grey dots, lines, circles, and the circular surface are the system-environment state spaces for the four cases. See the text for definitions of the mathematical symbols.

adaptive systems can learn to anticipate future environmental changes and respond proactively to counter sensory and response delays, either through precursor signals [16, 17], inner biological clocks [15, 56], or excitability adaptation [57, 58]. Such anticipatory mechanisms can lead to large fitness advantages. Below, we make this quantitative.

We model a periodically-switching environment as the deterministic limit of a switch with infinite intermediate Markov transitions as illustrated in Fig. 5(a). The state space of the environment is a ring, and the environment-system joint state space is now two rings, as shown in gray in Fig. 5(b). Since the parsing to marginal and conditional probabilities in Eqs. (2), (6), and (15) are valid generally, no matter if the environmental switching is stochastic or deterministic, our task here is to derive how the NEQ probability flux relates to the conditional probability difference for periodic environments. The results, in comparison to the Markov random environment, are summarized in Fig. 6 and explained as follows.

**The value of anticipation depends on the phase of the environment.** We first derive the fitness parsing in the pure switching case. For a switch $a \leftrightarrow b$ under a periodic environment, let us define $\mathcal{J}(\theta)$ as the net flux density from $b$ to $a$ at a given phase of the environment $\theta \in [0, T_A + T_B]$, along the blue horizontal edges in Fig. 5(b). Then, we can derive the following relation between net flux density and conditional probabilities (see Appendix G in SM [51] for derivation and discussion)

$$\pi_{a|A} - \pi_{a|B} = \mathcal{K} \circ \mathcal{J}, \tag{18}$$

where the contributions of the NEQ flux at different phases are superimposed with a linear kernel $\mathcal{K}$:

$$\mathcal{K} \circ \mathcal{J} = \int_0^{T_A} \left(\frac{1}{2} - \frac{\theta}{T_A}\right) \mathcal{J}(\theta) \mathrm{d}\theta \tag{19a}$$

$$+ \int_0^{T_B} \left(\frac{1}{2} - \frac{\theta}{T_B}\right) [-\mathcal{J}(T_A + \theta)] \mathrm{d}\theta. \tag{19b}$$

Similar to the Markov random environment case in Eq. (8), this tracking term sums contributions from the two environments. The difference is that the phase matters under periodic environments, so the two terms $\bar{T}_A J + \bar{T}_B J$ in the Markov random case are replaced by integration over the flux profiles respectively. Together with Eq. (6), this result shows the synchrony gain from fluxes for a two-state system in a periodic environment,

$$\pi_{Aa} - \pi_A \pi_a = \pi_{Bb} - \pi_B \pi_b = \pi_A \pi_B \, \mathcal{K} \circ \mathcal{J} \tag{20}$$

This linear kernel $\mathcal{K}$ demonstrates the benefit of anticipatory fluxes in a periodic environment: for the latter half of each environmental epoch ($\theta > T_A/2$, $\theta > T_B/2$), anticipatory net fluxes toward the currently less fit state ($a \to b$ in $A$ and $b \to a$ in $B$) contribute positively. Furthermore, this gain from anticipatory flux gets higher when $\theta$ is closer to the switching time $T_A$ or $T_B$. A toy example is presented in

Appendix H of SM [51] to show how late anticipatory flux helps the synchronization task and how anticipatory systems are more susceptible to switching errors.

There are, in fact, multiple mathematically-equivalent expressions of the kernel because the integration of net flux density $\mathcal{J}$ over the full cycle must be zero—there should be no net probability transport from $b$ to $a$ over the whole period. To illustrate this mathematical degeneracy, consider the simplest flux density with two Dirac delta function pulses: $\mathcal{J}(\theta) = J\delta(\theta - \theta_1) - J\delta(\theta - \theta_2)$. The two pulses must have the same strength to be integrated to zero over the whole period. These pulses are anticipatory if $0 \leq \theta_2 \leq T_A$ and $T_A \leq \theta_1 \leq T_A + T_B$. We then get $\mathcal{K} \circ \mathcal{J} = [\theta_2/T_A - 1/2]J + [(\theta_1 - T_A)/T_B - 1/2]J = [\theta_2/T_A + (\theta_1 - T_A)/T_B - 1]J$. This shows that the "1/2" factors can be replaced with any two constants $p, q$ that summed to one, $p + q = 1$: a mathematical degeneracy due to the two pulses having the same strength. However, physically, we expect the gain to be the same from employing the same type of anticipatory fluxes within the two environments. This "fixes the gauge" to the equal splitting $p = q = 1/2$ for physical interpretations. For example, if both pulses are located at 2/3 of each environmental epoch, i.e. $\theta_2 = 2T_A/3$ and $\theta_1 = T_A + 2T_B/3$, then the gains from both fluxes should be the same: in this case, both $(2/3 - 1/2)J = J/6$. In short, Eq. (20) gives the synchrony-flux relation under periodic environments with different kernels characterizing the benefits of well-timed anticipation.

**Kernel $\mathcal{K}$ captures the fitness gain of fluxes at different phases of the periodic environment.** The kernel $\mathcal{K}$ gives the fitness parsing for an adapting population with growth. For the adapting population, the system's state space is now a real line segment $[0, 1]$ instead of just two states $(a, b)$. The system-environment joint state space then becomes the two-dimensional curved side of the cylinder as shown at the lower-right of Fig. 6. We can then derive (see Appendix I of SM [51])

$$\langle f|A\rangle - \langle f|B\rangle = \mathcal{K} \circ \bar{\mathcal{J}} \; , \tag{21}$$

where $\bar{\mathcal{J}}(\theta) = \int_0^1 \mathcal{J}(f, \theta)\mathrm{d}f = \frac{\mathrm{d}\hat{f}(\theta)}{\mathrm{d}\theta}$ is the total flux across the system's state space at environmental phase $t$ and $\hat{f}(\theta)$ is the solution of the ODE of $f$ under a periodic environment. The fitness parsing can then be derived:

$$\mathrm{LTGR} = \bar{g}_a\langle f\rangle + \bar{g}_b\langle 1 - f\rangle + \pi_A\pi_B\,(\mathcal{K} \circ \bar{\mathcal{J}})\Delta_g. \tag{22}$$

This shows that (1) anticipatory fluxes can help tracking when well-timed (in the latter half of the environmental phase) and (2) the fitness gain from tracking also diminishes when the environment switches fast[8] ($T_A, T_B \to 0$) or when the environment is strongly biased ($\pi_A \to 0$ or 1 such that $\pi_A\pi_B \to 0$), similar to the random environment case.

[8] When $T \to 0$, the integral

$$\pm\int_0^T (1/2 - \theta/T)\mathcal{J}(\theta)\mathrm{d}\theta \sim \mp\mathcal{J}(0)\frac{1}{T}\int_0^T \theta\,\mathrm{d}\theta \to 0.$$

## EXAMPLE 5: HOW TO CONTROL ENVIRONMENTS TO MANIPULATE SYSTEMS

So far, we considered adaptive system degrees of freedom in unpredictable environments. Here, we consider the reverse: the system is given, and we can control the environment. We illustrate this with a toy model of drug resistance.

When drugs are applied to killing populations of pathogens, such as bacteria, cancer, or viruses, the resulting selective pressure leads the organism to become drug-resistant. A sufficiently strong treatment of a drug will drive the rise of a resistant mutant, and subsequent treatment will fail. Accordingly, drug resistance can be viewed as an evolutionary game that we drug-givers are playing against the disease pathogen [59]. An important successful example is "integrated pest management" [60, 61], where the problem of pesticide resistance has been controlled through better treatment schedules and other strategies explicitly designed to fight resistance. We now show how a toy model of drug resistance, similar to the one in [62], can be decomposed into components, to ultimately improve evolution-inspired treatment schedules.

Consider a pathogen with drug-sensitive population $N_S$ and drug-resistant population $N_R$. We assume that both subpopulations grow at rate $g$ without drug and that the sensitive one reduces to $g - \delta$ when the drug is on. We also assume that that switching from sensitive to resistant only happens under drug, with rate $r$, and re-sensitization happens only when there's no drug, with rate $s$. For a large population, the dynamics can be described by ODEs:

$$\dot{N}_S = (g - \delta D)\,N_S - rDN_S + s(1 - D)N_R \tag{23a}$$
$$\dot{N}_R = g\,N_R + rDN_S - s(1 - D)N_R \tag{23b}$$

where $D = 1$ or 0 represents drug on or off. As before, we then rewrite them in terms of the total population $N$ and the population fraction of drug-resistant cells $f$ for drug-on,

$$\dot{N} = [g - (1 - f)\,\delta]N; \tag{24a}$$
$$\dot{f} = f(1 - f)\,\delta + (1 - f)\,r, \tag{24b}$$

and drug off,

$$\dot{N} = g\,N \text{ and } \dot{f} = -s\,f. \tag{25}$$

Denoting $\pi_{\text{on}}$ as the time fraction of drug-on, Eqs. (15) and (22) gives us the population long-term growth rate under Markov stochastic or periodic drug on/off switching:

$$\mathrm{LTGR} = [g - \langle 1 - f\rangle\,\pi_{\text{on}}\,\delta] + (\text{tracking}) \tag{26}$$

where the tracking term depends on the switching environment being Markov random, $\pi_{\text{on}}(1 - \pi_{\text{on}})\,(\bar{T} \cdot \bar{J})\,\delta$,

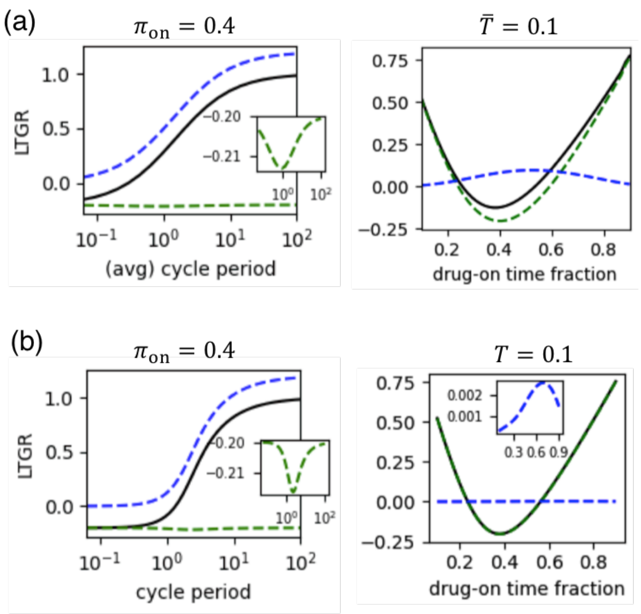

FIG. 7. **Fast environment switching reduces tracking gain whereas optimal environment time fraction reduces generalism gain.** **(a)** shows the total long-term population growth rate of drug sensitive or resistant cells under Markov random on/off drug switching. On the left, we vary the average cycle period $\bar{T} = \bar{T}_{\text{on}} + \bar{T}_{\text{off}}$ where $\bar{T}_{\text{on/off}}$ represents long-term average drug on/off intervals and fix the drug-on time fraction $\pi_{\text{on}} = \bar{T}_{\text{on}}/\bar{T}$. On the right, we vary $\pi_{\text{on}}$ and fix $\bar{T}$. **(b)** is similar to (a) but under periodic environment: $T = T_{\text{on}} + T_{\text{off}}$ where $T_{\text{on/off}}$ is the fixed interval of on/off and $\pi_{\text{on}} = T_{\text{on}}/T$. Parameters in both (a) and (b) are $g = 1, \delta = 5, r = 1, s = 3$. See Appendix J of SM [51] for how these are chosen. The time unit is chosen to be 100 days, i.e. rate quantities are per 100 days.

or periodic, $\pi_{\text{on}}(1-\pi_{\text{on}})\,(\mathcal{K}\circ\bar{\mathcal{J}})\,\delta$. We show below that the two different components—generalism and tracking—can be controlled by different environmental features.

On the one hand, when the drug on/off switching is fast, i.e. when the on-off cycle period is small, the tracking term diminishes as it has a prefactor of the cycle period. The long-term population growth rate is dominated by the generalism term. The plot on the left of Fig. 7 shows this for (a) Markov random environment and (b) periodic environment. Fast switching reduces the LTGR gain from tracking the environment, which originates from the growth rate/fitness difference of sensitive and resistant cells. Our results here for the one drug case are similar to the recommendation of Goldie, Coldman, and Gudauskas [63–65], that fast alternating non-cross-resistant chemotherapy every cycle in cancer should lead to the best response. Our parsing gives a new interpretation for how population re-sensitization to drug impacts the LTGR. The strategy of alternating drugs has been applied with notable successes [66, 67] and failures [68–70]. Of course, our Fig. 7 has only been made with one set of parameters in a toy model with only one drug and two phenotypes. We expect future generalizations of our result (to different parameter values and more realistic multi-state models) to explain the mixed clinical trial results and predict which dosing strategy will be optimal for inhibiting any given population.

On the other hand, there's an optimal drug on/off time fraction $\pi_{\text{on}}$ for killing the population as shown on the right of Fig. 7 (a) and (b). Eq. (26) shows that this optimality is due to the generalism. In the two limits of none or all drug-on, $\pi_{\text{on}} \to 0$ or 1, the tracking term approaches zero and the generalism term reaches its maximal value $g$ because $\langle f\rangle \to 1$ as $\pi_{\text{on}} = 1$ and both types have growth rate $g$ when there's no drug $\pi_{\text{on}} = 0$. For levels in between, $0 < \pi_{\text{on}} < 1$, both the tracking term and the $\langle 1-f\rangle\pi_{\text{on}}\delta$ term in the generalism term become positive, leading to the opposite bell shapes of the two components on the right of Fig. 7. The optimal level of $\pi_{\text{on}}$ increases if re-sensitization rate $s$ increases or resistant rate $r$ decreases (see Appendix J of SM [51]).

This example shows that the two components of LTGR are governed by different environmental features, and that fitness parsing helps interpret known results. The fast drug on/off switching primarily reduces the tracking gain whereas the optimal drug-on time fraction primarily reduces the generalism gain. The optimal levels of drug switching speed and on-time fraction are due to two distinct adaptive strategies—tracking and generalism.

## SUMMARY AND DISCUSSION

Parsing fitness into generalism and tracking components reveals key principles of adaptive dynamics: it explains optimal strategies like bet-hedging and phenotypic memory; clarifies why tracking gain diminishes in fast or biased environments, but can be boosted by well-timed anticipation in coherent ones; and shows how pathogenic populations can be optimally controlled by using fast environmental switching and optimal time-fractioning to independently suppress tracking and generalism.

We note two potential generalizations: extending the framework to multi-state systems or environments (Appendix C in SM [51]) and incorporating system-environment feedback mechanisms from finite resource or the system's active changes of environment [71, 72] like migration. We also note a conceptual parallel to neuroscience, where the distinction between generalism and tracking is analogous to that between rate and spike-time coding—a choice of strategy in sensory neurons that itself can depend on the stochasticity of the environment [73, 74].

## ACKNOWLEDGMENTS

We thank Gábor Balázsi for insightful feedback and Corey Weistuch for pointing us Ref. [62], which lead us to develop the model in Example 5. We are grateful for the financial support from the Laufer Center for Physical and Quantitative Biology at Stony Brook, the John Templeton Foundation (Grant ID 62564), and NIH (Grant RM1-GM135136).

## AUTHORS CONTRIBUTION

All authors conceptualized the project together. YJY developed the theory and derived the main results. CK validated and refined the results and developed the model used in Example 5. KD supervised all developments. All authors contributed equally to writing and editing the manuscript.

[1] A. Murugan, K. Husain, M. J. Rust, C. Hepler, J. Bass, J. M. J. Pietsch, P. S. Swain, S. G. Jena, J. E. Toettcher, A. K. Chakraborty, K. G. Sprenger, T. Mora, A. M. Walczak, O. Rivoire, S. Wang, K. B. Wood, A. Skanata, E. Kussell, R. Ranganathan, H.-Y. Shih, and N. Goldenfeld, Roadmap on biology in time varying environments, Phys. Biol. **18**, 041502 (2021), publisher: IOP Publishing.

[2] E. Levien, J. Min, J. Kondev, and A. Amir, Non-genetic variability in microbial populations: survival strategy or nuisance?, Rep. Prog. Phys. **84**, 116601 (2021).

[3] J. R. Bernhardt, M. I. O'Connor, J. M. Sunday, and A. Gonzalez, Life in fluctuating environments, Phil. Trans. R. Soc. B **375**, 20190454 (2020).

[4] E. Kussell and S. Leibler, Phenotypic Diversity, Population Growth, and Information in Fluctuating Environments, Science **309**, 2075 (2005).

[5] D. M. Wolf, V. V. Vazirani, and A. P. Arkin, Diversity in times of adversity: probabilistic strategies in microbial survival games, Journal of Theoretical Biology **234**, 227 (2005).

[6] M. C. Donaldson-Matasci, C. T. Bergstrom, and M. Lachmann, When Unreliable Cues Are Good Enough., The American Naturalist **182**, 313 (2013), publisher: The University of Chicago Press.

[7] M. K. Belete and G. Balázsi, Optimality and adaptation of phenotypically switching cells in fluctuating environments, Phys. Rev. E **92**, 062716 (2015).

[8] B. Xue, P. Sartori, and S. Leibler, Environment-to-phenotype mapping and adaptation strategies in varying environments, PNAS **116**, 13847 (2019).

[9] M. Acar, J. T. Mettetal, and A. van Oudenaarden, Stochastic switching as a survival strategy in fluctuating environments, Nat Genet **40**, 471 (2008), number: 4 Publisher: Nature Publishing Group.

[10] M. E. Grantham, C. J. Antonio, B. R. O'Neil, Y. X. Zhan, and J. A. Brisson, A case for a joint strategy of diversified bet hedging and plasticity in the pea aphid wing polyphenism, Biology Letters **12**, 20160654 (2016), publisher: Royal Society.

[11] J. R. Gremer, S. Kimball, and D. L. Venable, Within- and among-year germination in Sonoran Desert winter annuals: bet hedging and predictive germination in a variable environment, Ecology Letters **19**, 1209 (2016), _eprint: https://onlinelibrary.wiley.com/doi/pdf/10.1111/ele.12655.

[12] C. S. Maxwell and P. M. Magwene, When sensing is gambling: An experimental system reveals how plasticity can generate tunable bet-hedging strategies, Evolution **71**, 859 (2017).

[13] G. Lambert and E. Kussell, Memory and Fitness Optimization of Bacteria under Fluctuating Environments, PLOS Genetics **10**, e1004556 (2014), publisher: Public Library of Science.

[14] D. Nevozhay, R. M. Adams, E. Van Itallie, M. R. Bennett, and G. Balázsi, Mapping the Environmental Fitness Landscape of a Synthetic Gene Circuit, PLoS Comput Biol **8**, e1002480 (2012).

[15] T. Saigusa, A. Tero, T. Nakagaki, and Y. Kuramoto, Amoebae anticipate periodic events, Phys. Rev. Lett. **100**, 018101 (2008).

[16] I. Tagkopoulos, Y.-C. Liu, and S. Tavazoie, Predictive Behavior Within Microbial Genetic Networks, Science **320**, 1313 (2008), publisher: American Association for the Advancement of Science.

[17] A. Mitchell, G. H. Romano, B. Groisman, A. Yona, E. Dekel, M. Kupiec, O. Dahan, and Y. Pilpel, Adaptive prediction of environmental changes by microorganisms, Nature **460**, 220 (2009).

[18] K. Whitehead, M. Pan, K.-i. Masumura, R. Bonneau, and N. S. Baliga, Diurnally Entrained Anticipatory Behavior in Archaea, PLOS ONE **4**, e5485 (2009).

[19] S. E. Marzen and J. P. Crutchfield, Optimized bacteria are environmental prediction engines, Phys. Rev. E **98**, 012408 (2018), publisher: American Physical Society.

[20] M. L. Jabbur, B. P. Bratton, and C. H. Johnson, Bacteria can anticipate the seasons: Photoperiodism in cyanobacteria, Science **385**, 1105 (2024).

[21] S. Wang and L. Dai, Evolving generalists in switching rugged landscapes, PLOS Computational Biology **15**, e1007320 (2019), publisher: Public Library of Science.

[22] V. Sachdeva, K. Husain, J. Sheng, S. Wang, and A. Murugan, Tuning environmental timescales to evolve and maintain generalists, Proceedings of the National Academy of Sciences **117**, 12693 (2020).

[23] M. Slatkin, Hedging one's evolutionary bets, Nature **250**, 704 (1974), publisher: Nature Publishing Group.

[24] D. L. Venable, Bet Hedging in a Guild of Desert Annuals, Ecology **88**, 1086 (2007), _eprint: https://onlinelibrary.wiley.com/doi/pdf/10.1890/06-1495.

[25] A. J. Grimbergen, J. Siebring, A. Solopova, and O. P. Kuipers, Microbial bet-hedging: the power of being different, Current Opinion in Microbiology **25**, 67 (2015).

[26] J. Müller, B. A. Hense, T. M. Fuchs, M. Utz, and C. Pötzsche, Bet-hedging in stochastically switching environments, Journal of Theoretical Biology **336**, 144 (2013).

[27] A. Solopova, J. van Gestel, F. J. Weissing, H. Bachmann, B. Teusink, J. Kok, and O. P. Kuipers, Bet-hedging during bacterial diauxic shift, Proceedings of the National Academy of Sciences **111**, 7427 (2014), publisher: Proceedings of the National Academy of Sciences.

[28] A. Mayer, T. Mora, O. Rivoire, and A. M. Walczak, Diversity of immune strategies explained by adaptation to pathogen statistics, Proceedings of the National Academy of Sciences **113**, 8630 (2016), publisher: Proceedings of the National Academy of Sciences.

[29] P. G. Hufton, Y. T. Lin, T. Galla, and A. J. McKane, Intrinsic noise in systems with switching environments, Phys. Rev. E **93**,

052119 (2016).
[30] P. G. Hufton, Y. T. Lin, and T. Galla, Phenotypic switching of populations of cells in a stochastic environment, J. Stat. Mech. **2018**, 023501 (2018).
[31] L. Dinis, J. Unterberger, and D. Lacoste, Pareto-optimal trade-off for phenotypic switching of populations in a stochastic environment, J. Stat. Mech. **2022**, 053503 (2022), publisher: IOP Publishing and SISSA.
[32] A. Mayer, T. Mora, O. Rivoire, and A. M. Walczak, Transitions in optimal adaptive strategies for populations in fluctuating environments, Phys. Rev. E **96**, 032412 (2017), publisher: American Physical Society.
[33] A. Skanata and E. Kussell, Evolutionary Phase Transitions in Random Environments, Phys. Rev. Lett. **117**, 038104 (2016), publisher: American Physical Society.
[34] P. Patra and S. Klumpp, Emergence of phenotype switching through continuous and discontinuous evolutionary transitions, Phys. Biol. **12**, 046004 (2015), publisher: IOP Publishing.
[35] Y.-J. Yang and K. A. Dill, Deriving the Forces of Nonequilibria from Two Laws (2024), arXiv:2410.09277.
[36] Y.-J. Yang and K. A. Dill, Nonequilibrium Force-Flow Relations in Networks (2024), arXiv:2410.17495.
[37] U. Seifert, Entropy Production along a Stochastic Trajectory and an Integral Fluctuation Theorem, Phys. Rev. Lett. **95**, 040602 (2005).
[38] J. Wang, L. Xu, and E. Wang, Potential landscape and flux framework of nonequilibrium networks: Robustness, dissipation, and coherence of biochemical oscillations, PNAS **105**, 12271 (2008).
[39] M. Esposito and C. Van den Broeck, Three Detailed Fluctuation Theorems, Phys. Rev. Lett. **104**, 10.1103/PhysRevLett.104.090601 (2010).
[40] H. Ge and H. Qian, Physical origins of entropy production, free energy dissipation, and their mathematical representations, Phys. Rev. E **81**, 051133 (2010).
[41] Y.-J. Yang and Y.-C. Cheng, Potentials of continuous Markov processes and random perturbations, J. Phys. A: Math. Theor. **54**, 195001 (2021).
[42] Y.-J. Yang and H. Qian, Time-translational symmetry in statistical dynamics dictates Einstein relation, Green-Kubo formula, and their generalizations, Phys. Rev. E **107**, 024110 (2023).
[43] V. Mustonen and M. Lässig, Fitness flux and ubiquity of adaptive evolution, PNAS **107**, 4248 (2010).
[44] F. Zhang, L. Xu, K. Zhang, E. Wang, and J. Wang, The potential and flux landscape theory of evolution, J. Chem. Phys. **137**, 065102 (2012), publisher: American Institute of Physics.
[45] H. Qian, Fitness and entropy production in a cell population dynamics with epigenetic phenotype switching, Quant Biol **2**, 47 (2014).
[46] T. J. Kobayashi and Y. Sughiyama, Fluctuation Relations of Fitness and Information in Population Dynamics, Phys. Rev. Lett. **115**, 238102 (2015).
[47] A. Genthon and D. Lacoste, Fluctuation relations and fitness landscapes of growing cell populations, Sci Rep **10**, 11889 (2020), publisher: Nature Publishing Group.
[48] R. Rao and S. Leibler, Evolutionary dynamics, evolutionary forces, and robustness: A nonequilibrium statistical mechanics perspective, Proc. Natl. Acad. Sci. U.S.A. **119**, e2112083119 (2022).
[49] L. Van Valen, A new evolutionary law, Evolutionary Theory **1**, 1 (1973).
[50] P. Ao, Laws in Darwinian evolutionary theory, Physics of Life Reviews **2**, 117 (2005).
[51] See Supplementary Material at [URL] for derivations and discussions.
[52] D. Hartich, A. C. Barato, and U. Seifert, Stochastic thermodynamics of bipartite systems: transfer entropy inequalities and a Maxwell's demon interpretation, J. Stat. Mech. **2014**, P02016 (2014), publisher: IOP Publishing and SISSA.
[53] J. M. Horowitz and M. Esposito, Thermodynamics with Continuous Information Flow, Phys. Rev. X **4**, 031015 (2014).
[54] M. P. Leighton, J. Ehrich, and D. A. Sivak, Information Arbitrage in Bipartite Heat Engines, Phys. Rev. X **14**, 041038 (2024), publisher: American Physical Society.
[55] A. C. Barato, D. Hartich, and U. Seifert, Efficiency of cellular information processing, New J. Phys. **16**, 103024 (2014).
[56] Y. Mi, X. Liao, X. Huang, L. Zhang, W. Gu, G. Hu, and S. Wu, Long-period rhythmic synchronous firing in a scale-free network, PNAS **110**, E4931 (2013).
[57] Y. V. Pershin, S. La Fontaine, and M. Di Ventra, Memristive model of amoeba learning, Phys. Rev. E **80**, 021926 (2009).
[58] Y.-J. Yang, C.-C. Chen, P.-Y. Lai, and C. K. Chan, Adaptive synchronization and anticipatory dynamical systems, Phys. Rev. E **92**, 030701 (2015).
[59] A. Stein, M. Salvioli, H. Garjani, J. Dubbeldam, Y. Viossat, J. S. Brown, and K. Staňková, Stackelberg evolutionary game theory: how to manage evolving systems, Philosophical Transactions of the Royal Society B **378**, 20210495 (2023).
[60] C. J. Whelan and J. J. Cunningham, Resistance is not the end: lessons from pest management, Cancer Control **27**, 1073274820922543 (2020), publisher: SAGE Publications Inc.
[61] R. A. Gatenby and J. S. Brown, Integrating evolutionary dynamics into cancer therapy, Nature reviews Clinical oncology **17**, 675 (2020).
[62] M. Robertson-Tessi, J. S. Brown, M. I. Poole, M. Johnson, A. Marusyk, J. A. Gallaher, K. A. Luddy, C. J. Whelan, J. West, M. Strobl, V. Turati, H. Enderling, M. J. Schell, A. Tan, T. Boyle, R. Makanji, J. Farinhas, H. Soliman, D. Lemanne, R. A. Gatenby, D. R. Reed, A. R. A. Anderson, and C. H. Chung, Feasibility of an Evolutionary Tumor Board for Generating Novel Personalized Therapeutic Strategies (2023), pages: 2023.01.18.23284628.
[63] J. Goldie, A. Coldman, and G. Gudauskas, Rationale for the use of alternating non-cross-resistant chemotherapy, Cancer Treat Rep **66**, 439 (1982).
[64] M. Strobl, J. Gallaher, M. Robertson-Tessi, J. West, and A. Anderson, Treatment of evolving cancers will require dynamic decision support, Annals of Oncology **34**, 867 (2023).
[65] M. Perry, D. Doll, and C. Freter, *Perry's The Chemotherapy Source Book* (Wolters Kluwer Health, 2012).
[66] J. H. Glick, M. L. Young, D. Harrington, R. L. Schilsky, T. Beck, R. Neiman, R. I. Fisher, B. A. Peterson, and M. M. Oken, Mopp/abv hybrid chemotherapy for advanced hodgkin's disease significantly improves failure-free and overall survival: the 8-year results of the intergroup trial., Journal of clinical oncology **16**, 19 (1998).
[67] H. E. Grier, M. D. Krailo, N. J. Tarbell, M. P. Link, C. J. Fryer, D. J. Pritchard, M. C. Gebhardt, P. S. Dickman, E. J. Perlman, P. A. Meyers, *et al.*, Addition of ifosfamide and etoposide to standard chemotherapy for ewing's sarcoma and primitive neuroectodermal tumor of bone, New England Journal of Medicine **348**, 694 (2003).
[68] G. Bonadonna, M. Zambetti, and P. Valagussa, Sequential or alternating doxorubicin and cmf regimens in breast cancer with more than three positive nodes: ten-year results, Jama **273**, 542 (1995).

[69] J. Aisner, C. Cirrincione, M. Perloff, M. Perry, D. Budman, J. Abrams, L. Panasci, H. Muss, M. Citron, and J. Holland, Combination chemotherapy for metastatic or recurrent carcinoma of the breast–a randomized phase iii trial comparing caf versus vath versus vath alternating with cmfvp: Cancer and leukemia group b study 8281., Journal of clinical oncology **13**, 1443 (1995).

[70] S. De Placido, F. Perrone, C. Carlomagno, A. Morabito, C. Pagliarulo, R. Lauria, A. Marinelli, M. De Laurentiis, E. Varriale, G. Petrella, *et al.*, Cmf vs alternating cmf/ev in the adjuvant treatment of operable breast cancer. a single centre randomised clinical trial (naples gun-3 study), British journal of cancer **71**, 1283 (1995).

[71] K. Friston, The free-energy principle: a unified brain theory?, Nat Rev Neurosci **11**, 127 (2010).

[72] K. Friston, S. Samothrakis, and R. Montague, Active inference and agency: optimal control without cost functions, Biol Cybern **106**, 523 (2012).

[73] Z. F. Mainen and T. J. Sejnowski, Reliability of spike timing in neocortical neurons, Science **268**, 1503 (1995).

[74] T. Taillefumier and M. O. Magnasco, A phase transition in the first passage of a Brownian process through a fluctuating boundary with implications for neural coding, PNAS **110**, E1438 (2013).
